# Adverse childhood experiences are associated with illicit drug use among pregnant women with middle to high socioeconomic status: findings from the All Our Families Cohort

**DOI:** 10.1186/s12884-021-03591-1

**Published:** 2021-02-13

**Authors:** Cheryl L. Currie, Suzanne C. Tough

**Affiliations:** 1 Faculty of Health Sciences, University of Lethbridge, M3083 Markin Hall, 4401 University Drive, Lethbridge, Alberta T1K 3M4 Canada; 2 Cummings School of Medicine, University of Calgary, Calgary, Canada

**Keywords:** Adverse childhood experiences, Pregnancy, Illicit drug use, All our families

## Abstract

**Background:**

Adverse childhood experiences (ACEs) are associated with illicit drug use among pregnant women who are socioeconomically vulnerable. While it is assumed that the impact of ACEs on illicit drug use in pregnancy is reduced among women with higher socioeconomic status (SES), this assumption is not well tested in the literature. The objective of this study was to examine the impact of maternal ACEs on illicit drug use in a community-based sample of pregnant women with middle to high SES.

**Methods:**

This study is a secondary analysis of a prospective cohort study that collected data from 1660 women during and after pregnancy in Calgary, Canada between 2008 and 2011 using mailed surveys. Illicit drug use in pregnancy was self-reported by women at 34–36 weeks gestation. An established scale examined maternal ACEs before 18 years. Logistic regression models and 95% confidence intervals tested associations between maternal ACE scores and illicit drug use in pregnancy.

**Results:**

Overall, 3.1% of women in this predominantly married, well-educated, middle and upper middle income sample reported illicit drug use in pregnancy. Women with 2–3 ACEs had more than a two-fold increase, and women with 4 or more ACEs had almost a four-fold increase in illicit drug use in pregnancy, relative to women with 0–1 ACEs after adjustment for confounders. Exposure to child abuse was more consistently associated with illicit drug use in pregnancy than exposure to household dysfunction in childhood.

**Conclusions:**

Maternal ACEs were common and associated with a moderate increase in the odds of illicit drug use in pregnancy among Canadian women with middle to high SES.

## Background

The purpose of this study was to examine the role that adverse childhood experiences (ACE) could play in illicit drug use among pregnant women with moderate to high socioeconomic status (SES). Illicit (or street) drug use involves the use of substances manufactured and/or sold illegally. Although illicit drug use is reported by 1 to 6% of pregnant women and thus considered rare in most countries, when it does occur it can have serious consequences [1–8]. The four most commonly used illicit drugs in pregnancy are cannabis, cocaine, methamphetamine and opioids derived from street sources [9–11]. Systematic reviews have summarized the adverse impacts of these substances during and after pregnancy. Depending on the drug used, obstetrical complications include intrauterine growth restriction, placental abruption, preterm delivery, stillbirth, and maternal death [12–17]. Neonatal complications include low birth weight, congenital malformations, neonatal abstinence syndrome, and fetal death [15–18]. Prenatal exposure to cannabis, cocaine, methamphetamine, opioids, and other illicit drugs have also impact infant and child development, health, and academic achievement, including adverse impacts that extend well beyond school years [19–23].

Women who use illicit drugs while pregnant also take on risks that go beyond the specific drug used, as there is an inherent uncertainty about the dose, drug types, and contaminants ingested due to their undocumented origin [2, 24, 25]. For example, a 2018 study that asked illicit drug users to anonymously submit drug samples found 60% of the illicit Canadian drug supply was adulterated with fentanyl and other substances the person had not anticipated [26].

### ACEs and illicit drug use in pregnancy

Given illicit drug use in pregnancy has a plethora of adverse impacts on public health a strong research base is needed to inform prevention efforts. However, pregnant women who use illicit drugs are an elusive population who often choose to remain unidentified due to fear of reprisal and judgement. Hence, this population is difficult to study, and risk factors for illicit drug use in pregnancy are not well understood, particularly in non-clinical and more affluent populations [27, 28]. Research in general (i.e., non-pregnant) populations has highlighted adverse childhood experiences (ACEs) as particularly strong risk factors for illicit drug use among adults [29]. A 2017 systematic review of 37 studies found moderate to strong associations between an ACE score of four or more and a variety of health problems and at-risk behaviours in adulthood [29]. Across the 23 outcomes examined in this review, the strongest association was found between an ACE score of four or more and problematic drug use in adulthood (odds ratio above than seven) [29].

ACEs are defined as child maltreatment and exposure to household dysfunction before 18 years of age [30]. As social experiences, ACEs hamper a child’s ability to correctly attribute the intent of caregivers, trust, and develop secure attachments with them [31]. Over time, households characterized by abuse and neglect undermine a developing child’s ability to regulate their emotions and cope effectively with negative affect [31]. As a result, children and adolescents with elevated ACE scores display higher internalizing symptoms such as depression and anxiety, as well as externalizing symptoms such as substance use and other at-risk behaviours that may follow them into adulthood [32–37].

Given the large number of studies that have documented an association between ACE score and drug use in the general population, it is reasonable to expect that this association would extend into pregnancy. Yet, few studies have examined this association outside highly vulnerable groups. A US study found maternal ACEs of four or more were strongly associated with illicit drug use in pregnancy (odds ratio of six) within a sample of predominantly young, single women with low incomes and low educational attainment [38]. A German study examined the association between maternal child sexual abuse (CSA) and drug use among 255 pregnant women who were not socioeconomically vulnerable. They found two women who experienced CSA, and zero women who had not experienced CSA reported illicit drug use in pregnancy; thus an association could not be examined [39]. Studies with other samples that are not socioeconomically vulnerable have looked at associations between maternal ACEs and a combined substance use variable that includes drug use, alcohol use, and/or smoking in pregnancy, likely due to the low prevalence of illicit drug use in pregnancy [3, 40]. Thus, it remains unclear whether ACEs are associated specifically with illicit drug use in pregnancy among women who are not highly socioeconomically vulnerable [38]. This is important to understand, given potential increases in drug use during pregnancy due to the legalization of cannabis and the continued opioid crisis in Canada and other jurisdictions, and the need to understand the populations of pregnant women in which these increases may be anticipated.

The purpose of the present study was to address this gap by examining the association between maternal ACEs and illicit drug use in a moderately sized, community-based sample of pregnant women with middle to high socioeconomic status.

## Methods

### Study sample

The current investigation is a cross-sectional analysis of the All Our Families prospective cohort which collected data from women during and after pregnancy on determinants of maternal and infant health [41–43]. Pregnant women were recruited from medical offices and through laboratory services in Calgary, Canada between 2008 and 2011. Inclusion criteria were: maternal age ≥ 18 years, being < 25 weeks’ gestation, receiving prenatal care, and being fluent in English. The present analysis was conducted in 2020. The dataset supporting the conclusions of this article is available in the PolicyWise for Children & Families SAGE Metadata repository [S01–197845.4: https://sagemetadata.policywise.com/nada/index.php/catalog/1#metadata-identification] [43].

### Procedure

Pregnant women and mothers completed six mailed survey packages with postage-paid return envelopes spanning pregnancy to 3 years postpartum; three of which were used in this secondary analysis (mean time to complete: 25 min each) [41]. The first time point used in this analysis was collected at < 25 weeks gestation and included questions on participant sociodemographics. The second timepoint was collected at 34–36 weeks gestation and included questions on illicit drug use during pregnancy. Data for the third time point were collected 3 years after birth and included questions about maternal ACEs before 18 years of age. Questions about maternal ACEs were asked at the third data collection time point because this longitudinal study was developed and funded in phases, with data collection about maternal ACEs funded during the third data collection window. Written informed consent was obtained at each time point. Full data collection procedures are reported elsewhere [41, 44].

### Methods of follow-up

Approximately 83% of the 4011 pregnant women who were approached about the study met inclusion criteria and agreed to participate (*N* = 3341). Trained research assistants contacted the participants if data were missing or clarification of responses was required. Participants who failed to return their questionnaire within 3 weeks were contacted by telephone and/or e-mail and reminded to complete the questionnaire; multiple attempts were made until the participant was contacted and provided the opportunity for a repeat mail-out. To keep participants engaged and updated, congratulation cards were sent after the birth of their baby, as well as newsletters semi-annually containing project progress and preliminary results. Despite these efforts, there was attrition over the course of the study with approximately 70% of eligible participants returning all survey packages mailed to them [41, 44]. At the three-year time point 2909 women were eligible for follow-up. Among these, 60% completed all relevant questions related to the variables examined in the present analysis (*N* = 1680).

### Measures

#### Drug use

The use of illicit drugs in pregnancy was assessed at 34–36 weeks gestation by the question: *Since becoming pregnant (including before you knew you were pregnant), did you ever use illicit drugs*? Responses options were yes or no.

#### ACEs

At infant age 36 months, mothers were asked to recall ACEs that occurred in their lives before the age of 18 using a detailed questionnaire adapted from the original ACE checklist [45]. For consistency with the original scoring of Felitti et al. and in response to pilot testing, questions were simplified for some of the original ACE questions to elicit yes/no responses instead of frequencies (often/very often) during data collection [46]. Detailed information about specific ACE questions used in this study can be found in Table 1.

**Table 1:** Questions asked about adverse childhood experiences

The following questions are about events that happened during YOUR childhood; that is, before 18 years of age.	Response options
1. Did you live with anyone who was depressed, mentally ill, or suicidal?	Yes/no
2. Did you live with anyone who was a problem drinker or alcoholic?	Yes/no
3. Did you live with anyone who used illegal street drugs or who abused prescription medications?	Yes/no
4. Did you live with anyone who served time or was sentenced to serve time in prison, jail or other correctional facility?	Yes/no
5. Were your parents separated or divorced?	Yes/no
6. How often did your parents or adults in your household ever slap, hit, kick, punch or beat each other up?	Yes/no
7. Before age 18, how often did a parent or adult in your home ever hit, beat, kick, or physically hurt you in any way? (Please do not include spanking)	Never/once/ more than once
8. How often did a parent or adult in your home ever swear at you, insult you, or put you down?	Never/once/ more than once
9. How often did anyone at least 5 years older than you or an adult ever touch you sexually?	Never/once/ more than once
10. How often did anyone at least 5 years older than you or an adult try to make you touch them sexually?	Never/once/ more than once
11. How often did anyone at least 5 years older than you or an adult force you to have sex?	Never/once/ more than once

#### Sociodemographics

Maternal age, education, yearly before-tax household income, marital status, pregnancy intention, and parity (birth to a fetus > 24 weeks) were collected in the first questionnaire package completed by mothers at < 25 weeks gestation.

### Statistical analysis

Separate logistic regression models, using adjusted odds ratios (AORs) and 95% confidence intervals (CIs) assessed the odds of illicit drug use in pregnancy as a function of a 3-category ACE score (0–1 ACE, 2–3 ACEs and ≥ 4 ACEs), a 3-category child abuse score (no child abuse, 1 form of child abuse, 2 or more forms of child abuse); and childhood household dysfunction score (no household dysfunction, 1 form of household dysfunction, 2 or more forms of household dysfunction).

Given the goal of this analysis was to examine the impact of maternal ACEs on illicit drug use among pregnant women with middle to high SES, the small percentage of women living in households that earned less than the low income cut-off for a family of four during the period in which the data were collected (i.e., less than $40,000/yr) were removed from the analysis (*n* = 20, 1.2% of sample) [47]. Thus, the final sample size examined was *N* = 1660 pregnant women, all of whom had household incomes of $40,000/yr or more. Given there was variation in household income in the remaining sample, household income was adjusted for as a covariate. Other variables associated with illicit drug use in pregnancy at *p* < 0.20 were retained as confounders in regression models including maternal age, marital status, education, and whether the pregnancy was intended.

We purposively refrained from controlling for mental health given the impacts of ACE score on mental health are well documented across longitudinal studies; as are the impacts of mental health on illicit drug use [29]. Thus, controlling for mental health, which likely sit on the causal pathway between ACEs and the use of illicit drugs in pregnancy, would introduce bias by decomposing the total effect of x on y into its constitute parts [48]. A 95% confidence interval (CI) was selected a priori given 1 to 6% of pregnant women report illicit drug use in community-based samples. Thus statistical power was expected to be low despite a moderately sized sample of 1660 pregnant women, and use of a 99% CI may have resulted in a Type II error [1–8]. Missing data were handled using listwise deletion. Data were examined using SPSS 26.0.

## Results

### Sample sociodemographics

Participants ranged in age from 18 to 45 years at < 25 weeks gestation (M = 30.9 years, SD = 4.3, range 18 to 45 years). As shown in Table 2, most were married (90.2%) or living common law (6.1%). The current pregnancy was intended for approximately 85% of the sample. Women were highly educated with 81.1% having completed university or college. All lived in at least middle income households. Approximately two thirds of the sample had household incomes that exceeded the median for households with children in Canada ($95,900) during the time frame in which the data were collected [49]. This included a third of the sample who reported incomes at or above $150,000 per year, which placed them in the top 20% of earners for households with children in Canada [49].

**Table 2:** Overall sample characteristics and prevalence of illicit drug use in pregnancy by sample characteristic (*N* = 1660)

Maternal characteristic	Sample frequency *n* (%)	Frequency of illicit drug use in pregnancy by characteristic *n* (%)
Sample total	1660 (100)	51 (100)
Age		
< 35 yrs	1328 (80.0)	suppressed ^a^
≥ 35 yrs	332 (20.0)	suppressed
Marital status		
Married	1497 (90.2)	34 (2.3)
Living common law	101 (6.1)	suppressed
Single	62 (3.7)	suppressed
Education		
Less than a post-secondary degree	313 (18.9)	24 (7.7)
Post-secondary degree	1347 (81.1)	27 (2.0)
Household income (yearly)		
$40,000 – $99,999	555 (33.4)	26 (4.7)
$100,000 - $149,999	546 (32.9)	15 (2.7)
≥ $150,000	559 (33.7)	10 (1.8)
Parity		
No previous births	816 (49.5)	37 (4.5)
≥ 1 previous birth	833 (50.5)	14 (1.7)
Pregnancy was intended		
Yes	1408 (84.8)	26 (1.8)
No	252 (15.2)	25 (9.9)
ACE score		
0–1	1023 (61.6)	14 (1.4)
2–3	403 (24.3)	20 (5.0)
≥ 4	234 (14.1)	17 (7.3)
Maternal ACE child abuse score		
No child abuse	930 (56.1)	15 (1.6)
1 form of child abuse	435 (26.2)	18 (4.1)
2–3 forms of child abuse	294 (17.7)	18 (6.1)
Maternal ACE household dysfunction score		
No household dysfunction	864 (52.0)	15 (1.7)
1 form of household dysfunction	393 (23.7)	14 (3.6)
2–6 forms of household dysfunction	403 (24.3)	22 (5.5)

^a^ Data suppressed due to low cell count (under *n* = 10)

### Maternal ACE score and illicit drug use in pregnancy

Maternal ACE score ranged from 0 to 8 (M = 1.5, SD = 1.7). ACE exposures were common among expectant mothers with 62% reporting at least one ACE before the age of 18. Four or more ACEs were reported by 14.1% of the sample. Overall, 3.1% of the sample reported illicit drug use during their current pregnancy (*n* = 51). As shown in Table 2, this included 1.4% of women with 0–1 ACEs reported illicit drug use in pregnancy, 5% of women with 2–3 ACEs, and 7.3% of women with 4 or more ACEs. In an adjusted logistic regression model, the association between ACE score and illicit drug use in pregnancy was moderate in strength. Compared to women with 0–1 ACEs, women with 2–3 ACEs had more than a three-fold increase, and women with 4 or more ACEs had almost a four-fold increase in the odds of illicit drug use in pregnancy after adjustment for confounders.

Child abuse was reported by 43.7% of the sample. Emotional abuse was most commonly reported (35.5%), followed by physical abuse (16.7%) and sexual abuse (13.3%). Compared to women who did not experience child abuse, women who experienced 1 form of child abuse had more than a two-fold increase in the odds of illicit drug use in pregnancy; while women who experienced 2 to 3 forms of child abuse had almost a three-fold increase in the odds of illicit drug use in pregnancy after adjustment for confounders (Table 3).

**Table 3:** Odds ratios (ORs), adjusted odds ratios (AORs), and 95% confidence intervals for illicit drug use in pregnancy by ACE category (*N* = 1660)^a^

Models	OR (95% CI)	AOR (95% CI)
Model 1: Maternal ACE score < 18 years		
0–1 ACEs	1.0 (Reference)	1.0 (Reference)
2–3 ACEs	**3.8 (1.9, 7.5)**	**3.0 (1.5, 6.1)**
≥ 4 ACEs	**5.7 (2.7, 11.6)**	**3.7 (1.7, 8.0)**
Model 2: Maternal ACE child abuse score < 18 years		
No abuse in childhood	1.0 (Reference)	1.0 (Reference)
1 form of child abuse	**2.6 (1.3, 5.3)**	**2.3 (1.1, 4.7)**
2–3 forms of child abuse	**4.0 (2.0, 8.0)**	**2.8 (1.3, 5.7)**
Model 3: Maternal ACE household dysfunction score < 18 years		
No household dysfunction	1.0 (Reference)	1.0 (Reference)
1 form of household dysfunction	2.1 (1.0, 4.4)	1.5 (0.7, 3.2)
2–6 forms of household dysfunction	**3.3 (1.7, 6.4)**	**2.2 (1.1, 4.4)**

^a^ Variables significant using a 95% CI are presented in bold. Models were adjusted for maternal age, education, income, marital status, and whether the pregnancy was intended

At least one form of household dysfunction in childhood was reported by 48.0% of the sample. The most common exposure was mental illness in the home (24.6%) followed by parental separation or divorce (22.8%), and parental substance abuse (20.8%). There was no association between experiencing one form of household dysfunction in childhood and illicit drug use in pregnancy. As shown in Table 3, approximately one quarter of the sample reported 2–6 forms of household dysfunction in childhood. This subsample had more than a two-fold increase in the odds of illicit drug use in pregnancy.

## Discussion

Maternal ACEs were common in this study, and significantly associated with illicit drug use in this community-based, middle to high-income sample of pregnant women. The proportion of the sample that reported illicit drug use while pregnant was 3.1%, which is comparable to other studies which show that 1 to 6% of women in community-based samples report illicit drug use in pregnancy [1–7]. The frequency of ACEs reported in this study was high, but comparable to national US research collected within a similar time frame. Specifically, 62% of women in the present study reported at least one ACE, and a mean ACE score of 1.5. A 2011–2014 surveillance study across 23 US states similarly documented that 62% of adults reported at least one ACE, and a mean ACE score of 1.7 [50]. The US survey found childhood emotional, physical and sexual abuse was reported by 34, 18, and 16% of American women; respectively [50]. In the present Canadian sample, childhood emotional, physical and sexual abuse was reported by 36, 17, and 13% of women; respectively. Exposure to household dysfunction in the present sample was also similar to US estimates, with parental separation/divorce and parental substance use reported by 34 and 18% of American women, compared to 23 and 21% of Canadian women in this study [50].

In the present study, an ACE score of four or more was *moderately* associated with illicit drug use in pregnancy (odds ratio 3.7). This finding differs from Chung et al. (2010) who found four or more ACEs was *strongly* associated with illicit drug use in pregnancy (odds ratio above 7) among women in the US [38]. A key reason for this difference may be sociodemographic differences between the samples. In the US study, the sample largely consisted of young (mean age: 24 years), single (75% unmarried) women with low educational attainment (18% had completed university or college) and low income [38]. In the present study, the sample largely consisted of mature (mean age: 31 years), married (90%), well educated (81% had completed university or college) women with middle to high household incomes. Higher SES and/or access to universal health care among women in the Canadian, as compared to the US sample, may have provided some level of protection against the impacts of maternal ACEs on illicit drug use in pregnancy. For example, most women in our sample had a post-secondary degree and may have been more aware of the impacts that ACEs could have on their well-being and/or the impacts that illicit drug use could have on their pregnancy than women examined in the Chung et al. study. Universal access to prenatal care in the Canadian sample may have resulted in increased contact with medical providers who could inform women about the dangers of substance use in pregnancy, and who could refer them to mental health resources if they were struggling with childhood trauma or substance use. The moderate to high SES of the sample also meant that women had the financial means to access mental health resources for childhood trauma and/or substance use that are not typically covered by health insurance. We note that there are only a small number of effective therapies for substance use in pregnancy [51]. These primarily involve behavioural counselling, the costs of which are seldom covered by government health insurance programs, and that can require significant personal resources to take part in [51].

Yet, it is also important to note that the elevated SES of the present sample did not eliminate the impact of ACEs on illicit drug use in pregnancy. The association was statistically significant and moderate in strength, suggesting that even among more affluent populations, maternal ACEs are an important risk factor for illicit drug use in pregnancy. Mediators of this association may be similar to those observed in men and women in general population studies. It is well documented that exposure to ACEs may result in psychological, behavioral and neurobiological adaptations that promote short-term survival for a child in their environment, while conferring longer-term vulnerability across a wide range of health-risk behaviors, including drug use [52]. ACEs result in threat-related social information processing biases, heightened emotional reactivity, difficulties with emotional regulation, and blunted reward responsivity which can drive individuals toward more intense reward-seeking in order to successfully alleviate these adverse states [53–57]. Entering into pregnancy with an elevated ACE score may heighten these impacts given it is an emotionally vulnerable time for women. From a multigenerational perspective, women with elevated ACE scores may also receive less social support from their parents during pregnancy relative to other women, or may continue to experience emotional abuse from their parents as adults, which could influence or exacerbate illicit drug use in pregnancy. It is recommended that future studies examine these and other potential mediators of the associations observed in this study in large samples of pregnant women in order to accommodate such analyses. The findings of this study do not suggest that a large percentage of women with an elevated ACE score will use illicit drugs in pregnancy. The overall percentage of women who reported illicit drug use while pregnant remained small, regardless of maternal ACE exposure. Indeed, even among women with 4 or more ACEs, 93% reported they did not use illicit drugs during their pregnancy. Building on the work of Hall & van Teijlingen (2006), more qualitative studies with pregnant women who use illicit drugs is recommended to understand their needs and inform prevention efforts [58]. The results of this study suggest these qualitative samples should include community-based women from across the socioeconomic spectrum to ensure a full understanding of the drivers of illicit drug use in pregnancy across different populations.

### Limitations

The associations documented in this observational study do not imply causation. Data collected on maternal ACEs and illicit drug use in pregnancy were based on self-report. While retrospective reports of major, easily defined ACEs have acceptable psychometric properties [59, 60], illicit drug use in pregnancy is frequently underreported [5]. We note that underreporting is particularly amplified in jurisdictions that have laws that penalize women for prenatal drug use [4–6], which was not the case for the present sample. Data were collected in a Canadian province that does not have laws that penalize women for prenatal drug use. Underreporting due to social desirability bias is also a concern [61]. To reduce this data were collected by mailed surveys rather than face-to-face interviews, women were reminded their responses were confidential, and returned surveys included only a participant’s ID [62, 63]. It was also made clear to participants that their responses would not be shared with clinical providers or become part of their medical record, that all research staff who reviewed their survey responses had signed confidentiality agreements, that their names would not be kept in the same database as their survey responses, and that the project had been reviewed by the Privacy Commissioner to ensure the highest protection of the information they shared. Despite these efforts, the likely underreporting of illicit drug use in pregnancy remains a limitation of this study. That said, the literature does not suggest differential reporting of substance use in pregnancy by maternal ACE score. Thus, we expect the misclassification of some pregnant women who had used illicit drugs into the non-drug use group was non-differential and would not bias the associations observed in the direction of a Type 1 error [64]. We note there was attrition over the course of the study, with approximately 70% of participants returning all survey packages mailed to them, and 58% of women completing all questions relevant to this secondary analysis of the data.

English fluency was required to participate. Census data indicate 92% of Alberta adults across all ages were fluent in English during the period in which data were collected [65]. Given English fluency is higher in younger populations, and no participants exceeded the age of 45 in this study, we do not expect English fluency significantly impacted participant recruitment, but note it as a possible limitation.

### Conclusions

Illicit drug use in pregnancy is a critical public health concern linked with a variety of harmful maternal and fetal consequences. In the present study, maternal ACEs were common and associated with a moderate increase in illicit drug use among community-based pregnant women with middle to high SES in Canada. The present findings speak to the public health significance of maternal ACEs on illicit drug use in pregnancy, and the need for increased resources to support women of childbearing age who have experienced childhood adversity.

## Data Availability

The dataset supporting the conclusions of this article is available in the PolicyWise for Children & Families SAGE Metadata repository [S01–197845.4: https://sagemetadata.policywise.com/nada/index.php/catalog/1#metadata-identification] [43].

## Abbreviations

ACEs: Adverse childhood experiences
AOR: Adjusted odds ratio
CSA: Child sexual abuse
CI: Confidence interval
OR: Odds ratio
SES: Socioeconomic status

## References

[1] Kocherlakota P (2014). Neonatal abstinence syndrome. Pediatrics..

[2] Cook JL, Green CR, de la Ronde S, Dell CA, Graves L, Ordean A (2017). Epidemiology and effects of substance use in pregnancy. J Obstet Gynaecol Canada.

[3] Smith MV, Gotman N, Yonkers KA (2016). Early childhood adversity and pregnancy outcomes. Matern Child Health J.

[4] Kotelchuck M, Cheng ER, Belanoff C, Cabral HJ, Babakhanlou-Chase H, Derrington TM (2017). The prevalence and impact of substance use disorder and treatment on maternal obstetric experiences and birth outcomes among singleton deliveries in Massachusetts. Matern Child Health J.

[5] Chiandetti A, Hernandez G, Mercadal-Hally M, Alvarez A, Andreu-Fernandez V, Navarro-Tapia E (2017). Prevalence of prenatal exposure to substances of abuse: questionnaire versus biomarkers. Reprod Health.

[6] Ko JY, Patrick SW, Tong VT, Patel R, Lind JN, Barfield WD (2016). Incidence of neonatal abstinence syndrome - 28 States, 1999–2013. Morb Mortal Wkly Rep.

[7] Lester BM, Tronick EZ, LaGasse L, Seifer R, Bauer CR, Shankaran S (2002). The maternal lifestyle study: effects of substance exposure during pregnancy on neurodevelopmental outcome in 1-month-old infants. Pediatrics..

[8] Administration SA and MHS (2013). Results from the 2012 National Survey on Drug Use and Health: Detailed tables.

[9] Krans EE, Patrick SW (2016). Opioid use disorder in pregnancy. Obstet Gynecol.

[10] Mcmillin GA, Wood KE, Strathmann FG, Krasowski M (2015). Patterns of drugs and drug metabolites observed in meconium. Ther Drug Monit.

[11] Roncero C, Valriberas-Herrero I, Mezzatesta-Gava M, Villegas JL, Aguilar L, Grau-López L (2020). Cannabis use during pregnancy and its relationship with fetal developmental outcomes and psychiatric disorders: a systematic review. Reprod Health.

[12] Warner TD, Roussos-Ross D, Behnke M (2014). It’s not your mother’s marijuana: effects on maternal-fetal health and the developing child. Clin Perinatol.

[13] dos Santos JF, de Melo Bastos Cavalcante C, Barbosa FT, Gitaí DLG, Duzzioni M, Tilelli CQ (2018). Maternal, fetal and neonatal consequences associated with the use of crack cocaine during the gestational period: a systematic review and meta-analysis. Arch Gynecol Obstet.

[14] Gouin K, Murphy K, Shah PS (2011). Effects of cocaine use during pregnancy on low birthweight and preterm birth: Systematic review and metaanalyses. Am J Obstet Gynecol.

[15] Kalaitzopoulos D-R, Chatzistergiou K, Amylidi A-L, Kokkinidis DG, Goulis DG (2018). Effect of methamphetamine hydrochloride on pregnancy outcome. J Addict Med.

[16] Lind JN, Interrante JD, Ailes EC, Gilbosa S, Khan S, Frey M (2017). Maternal use of opioids during pregnancy and congenital malformations: a systematic review. Am Acad Pediatr.

[17] Gunn JKL, Rosales CB, Center KE, Nuñez A, Gibson SJ, Christ C (2016). Prenatal exposure to cannabis and maternal and child health outcomes: a systematic review and meta-analysis. BMJ Open.

[18] Stover MW, Davis JM (2015). Opioids in pregnancy and neonatal abstinence syndrome. Semin Perinatol.

[19] Porath A, Beirness D, Diplock J, Kalant H. Clearing the smoke on cannabis: maternal cannabis use during pregnancy – an update. Ottawa: Canadian Centre on Substance Abuse; 2015.

[20] Richardson GA, Goldschmidt L, Larkby C, Day NL (2013). Effects of prenatal cocaine exposure on child behavior and growth at 10 years of age. Neurotoxicol Teratol.

[21] Diaz SD, Smith LM, Lagasse LL, Derauf C, Newman E, Shah R (2014). Effects of prenatal methamphetamine exposure on behavioral and cognitive findings at 7.5 years of age. J Pediatr.

[22] Conradt E, Flannery T, Aschner JL, Annett RD, Croen LA, Duarte CS, Friedman AM, Guille C, Hedderson MM, Hofheimer J, Jones MR, Ladd-Acosta C, McGrath M, Moreland A, et al. Prenatal opioid exposure: neurodevelopmental consequences and future research priorities. Pediatrics. 2019;144(3):e20190128. 10.1542/peds.2019-0128 PMC6759228 31462446

[23] Yeoh SL, Eastwood J, Wright IM, Morton R, Melhuish E, Ward M (2019). Cognitive and motor outcomes of children with prenatal opioid exposure: a systematic review and meta-analysis. JAMA Netw Open.

[24] Gonçalves H, Soares ALG, Ribeiro CG, Bierhals IO, Vieira LS, Santos APG dos (2016). Adverse childhood experiences and consumption of alcohol, tobacco and illicit drugs among adolescents of a Brazilian birth cohort. Cad Saude Publica.

[25] Varner MW, Silver RM, Hogue CJR, Willinger M, Parker CB, Thorsten VR (2014). Association between stillbirth and illicit drug use and smoking during pregnancy. Obstet Gynecol.

[26] Tupper KW, McCrae K, Garber I, Lysyshyn M, Wood E (2018). Initial results of a drug checking pilot program to detect fentanyl adulteration in a Canadian setting. Drug Alcohol Depend.

[27] Hans SL (1999). Demographic and psychosocial characteristics of substance-abusing pregnant women. Clin Perinatol.

[28] Wendell AD (2013). Overview and epidemiology of substance abuse in pregnancy. Clin Obstet Gynecol.

[29] Hughes K, Bellis MA, Hardcastle KA, Sethi D, Butchart A, Mikton C (2017). The effect of multiple adverse childhood experiences on health: a systematic review and meta-analysis. Lancet Public Heal..

[30] Dube SR, Felitti VJ, Dong M, Giles WH, Anda RF (2003). The impact of adverse childhood experiences on health problems: evidence from four birth cohorts dating back to 1900. Prev Med (Baltim).

[31] Petersen AC, Joseph J, Feit M (2014). New directions in child abuse and neglect research.

[32] Enns MW, Cox BJ, Asmundson GJG, Stein MB, Sareen J, Afifi TO (2008). Population attributable fractions of psychiatric disorders and suicide ideation and attempts associated with adverse childhood experiences. Am J Public Health.

[33] McDonald SW, Madigan S, Racine N, Benzies K, Tomfohr L, Tough S (2019). Maternal adverse childhood experiences, mental health, and child behaviour at age 3: the all our families community cohort study. Prev Med (Baltim)..

[34] Hunt TKA, Slack KS, Berger LM (2017). Adverse childhood experiences and behavioral problems in middle childhood. Child Abuse Negl.

[35] Dube SR, Anda RF, Felitti VJ, Edwards VJ, Williamson DF (2002). Exposure to abuse, neglect, and household dysfunction among adults who witnessed intimate partner violence as children: implications for health and social services. Violence Vict.

[36] Raposo SM, Mackenzie CS, Henriksen CA, Afifi TO (2014). Time does not heal all wounds: older adults who experienced childhood adversities have higher odds of mood, anxiety, and personality disorders. Am J Geriatr Psychiatry.

[37] Currie CL (2006). Animal cruelty by children exposed to domestic violence. Int J Child Abus Negl.

[38] Chung EK, Nurmohamed L, Mathew L, Elo IT, Coyne JC, Culhane JF (2010). Risky health behaviors among mothers-to-be: the impact of adverse childhood experiences. Acad Pediatr.

[39] Leeners B, Rath W, Block E, Görres G, Tschudin S (2014). Risk factors for unfavorable pregnancy outcome in women with adverse childhood experiences. J Perinat Med.

[40] Young-Wolff KC, Alabaster A, McCaw B, Stoller N, Watson C, Sterling S (2019). Adverse childhood experiences and mental and behavioral health conditions during pregnancy: the role of resilience. J Women’s Heal.

[41] Tough SC, McDonald SW, Collisson BA, Graham SA, Kehler H, Kingston D (2017). Cohort profile: the all our babies pregnancy cohort (AOB). Int J Epidemiol.

[42] Gracie SK, Lyon AW, Kehler HL, Pennell CE, Dolan SM, McNeil DA (2010). All our babies cohort study: recruitment of a cohort to predict women at risk of preterm birth through the examination of gene expression profiles and the environment. BMC Pregnancy Childbirth..

[43] Tough S, Benzies K, Collisson B, Graham S, McDonald S, Slater D, et al. All Our Babies/Families (AOB/F). V15 ed. Tough S, Benzies K, Collisson B, Graham S, McDonald S, Slater D, et al., editors. UAL Dataverse; Available from: 10.7939/DVN/10793

[44] McDonald SW, Lyon AW, Benzies KM, McNeil DA, Lye SJ, Dolan SM (2013). The All Our Babies pregnancy cohort: design, methods, and participant characteristics. BMC Pregnancy Childbirth.

[45] Felitti VJ, Anda RF, Nordenberg D, Williamson DF, Spitz AM, Edwards V (1998). Relationship of childhood abuse and household dysfunction to many of the leading causes of death in adults: the adverse childhood experiences (ACE) study. Am J Prev Med.

[46] Hetherington E, Racine N, Madigan S, McDonald S, Tough S. Relative contribution of maternal adverse childhood experiences to understanding children’s externalizing and internalizing behaviours at age 5: findings from the All Our Families Cohort. C Open. 2020;8(2):E352–9. 10.9778/cmajo.20190149 PMC7207036 32381686

[47] Statistics Canada (2016). Table 3.2 Low-income measures thresholds (LIM-AT, LIM-BT and LIM-MI) for households of Canada, 2010 - National Household Survey (NHS) Dictionary.

[48] Rohrer JM (2018). Thinking clearly about correlations and causation: graphical causal models for observational data. Adv Methods Pract Psychol Sci.

[49] Statistics Canada. 2011 National Household Survey. Ottawa: Statistics Canada catalogue no. 99-014-X2011047; 2013.

[50] Merrick MT, Ford DC, Ports KA, Guinn AS (2018). Prevalence of adverse childhood experiences from the 2011-2014 behavioral risk factor surveillance system in 23 states. JAMA Pediatr.

[51] Forray A (2016). Substance use during pregnancy. F1000Research..

[52] Duffy KA, McLaughlin KA, Green PA (2018). Early life adversity and health-risk behaviors: proposed psychological and neural mechanisms. Ann N Y Acad Sci.

[53] McLaughlin KA, DeCross SN, Jovanovic T, Tottenham N (2019). Mechanisms linking childhood adversity with psychopathology: learning as an intervention target. Behav Res Ther.

[54] Dennison MJ, Rosen ML, Sambrook KA, Jenness JL, Sheridan MA, McLaughlin KA (2019). Differential associations of distinct forms of childhood adversity with neurobehavioral measures of reward processing: a developmental pathway to depression. Child Dev.

[55] Brenhouse H, Lukkes J, Andersen S (2013). Early life adversity alters the developmental profiles of addiction-related prefrontal cortex circuitry. Brain Sci.

[56] Audrain-McGovern J, Rodriguez D, Leventhal AM, Cuevas J, Rodgers K, Sass J (2012). Where is the pleasure in that? Low hedonic capacity predicts smoking onset and escalation. Nicotine Tob Res.

[57] Herringa RJ, Birn RM, Ruttle PL, Burghy CA, Stodola DE, Davidson RJ (2013). Childhood maltreatment is associated with altered fear circuitry and increased internalizing symptoms by late adolescence. Proc Natl Acad Sci.

[58] Hall JL (2006). Van Teijlingen ER. A qualitative study of an integrated maternity, drugs and social care service for drug-using women. BMC Pregnancy Childbirth.

[59] Hardt J, Sidor A, Bracko M, Egle UT (2006). Reliability of retrospective assessments of childhood experiences in Germany. J Nerv Ment Dis.

[60] Reuben A, Moffitt TE, Caspi A, Belsky DW, Harrington H, Schroeder F (2016). Lest we forget: comparing retrospective and prospective assessments of adverse childhood experiences in the prediction of adult health. J Child Psychol Psychiatry.

[61] Tourangeau R, Yan T. Sensitive questions in surveys. Psychol Bull. 2007;133(5):859–83. 10.1037/0033-2909.133.5.859. 10.1037/0033-2909.133.5.859 17723033

[62] Krumpal I (2013). Determinants of social desirability bias in sensitive surveys: a literature review. Quality Quantity.

[63] Tourangeau R, Yan T (2007). Sensitive questions in surveys. Psychol Bull.

[64] Webb P, Bain C, Page A (2020). Essential Epidemiology.

[65] Statistics Canada (2017). Focus on geography series, 2016 census.

